# Determination of water permeability in stress-free lipid membranes through time-resolved small-angle neutron scattering

**DOI:** 10.1107/S1600576726007788

**Published:** 2026-09-25

**Authors:** Moritz P. K. Frewein, Ursula Perez-Salas, O. H. Samuli Ollila, Georg Pabst, Lionel Porcar

**Affiliations:** a Institut Laue–Langevin, 71 Avenue des Martyrs, 38042 Grenoble, France; b Department of Chemistry and iNANO, Aarhus University, Aarhus, Denmark; c Biophysics, Institute of Molecular Biosciences, University of Graz, NAWI Graz, 8010 Graz, Austria; d Physics Department, University of Illinois at Chicago, Chicago, IL 60607, USA; e VTT Technical Research Centre of Finland, 02044 Espoo, Finland; f Institute of Biotechnology University of Helsinki Viikinkaari 1 (PO Box 65) 00014 Helsinki Finland; Australian Centre for Neutron Scattering, ANSTO, Australia

**Keywords:** lipid membranes, permeation, small-angle neutron scattering, SANS, molecular dynamics, MD simulations, kinetics

## Abstract

Accurate quantification of water permeation across lipid membranes is challenging, with reported permeability values varying widely due to methodological differences and sample heterogeneity. Here, we show that time-resolved small-angle neutron scattering with rapid contrast variation enables direct non-invasive measurement of water permeation kinetics and membrane structure under iso-osmotic conditions. Our results indicate much faster water permeation in gel-phase bilayers than previously reported, likely due to improved structural control and the removal of multilamellar artefacts. Molecular-dynamics simulations agree in activation energies, though absolute permeabilities are higher *in silico*, highlighting methodological sensitivities. Our integrated approach offers new possibilities for future studies of diffusive solute transport across lipid membranes.

## Abstract 2

Time-resolved small-angle neutron scattering with rapid contrast variation enables direct non-invasive measurement of water permeation kinetics and membrane structure under iso-osmotic conditions for lipid membranes.

## 1. Introduction

Biological membranes are essential for compartmentalizing biochemical processes and regulating the selective exchange of nutrients and metabolites. Molecule transport across membranes occurs through several mechanisms: facilitated transport along concentration gradients via protein channels or carriers, active transport against concentration gradients via transporters or pumps and passive diffusion of solutes directly through the lipid bilayer (Yang & Hinner, 2015 ▸).

Passive diffusion through lipid bilayers requires the solute to overcome an activation free energy barrier to enter the membrane. This process is influenced by the physicochemical properties of the solute, such as size, charge and polarity, as well as the structural and compositional characteristics of the lipid bilayer itself (Yang & Hinner, 2015 ▸). Traditionally, passive diffusion is described by the solubility-diffusion model, which relates membrane permeability *P* to factors such as the solute’s partition coefficient into the membrane (Overton’s rule) and membrane thickness (Frallicciardi *et al.*, 2022*b* ▸). An alternative theory suggests that permeability is rather correlated with the area per lipid than the membrane thickness (Nagle *et al.*, 2008 ▸).

Among solutes, the permeability of membranes to water has received particular attention. Experimental studies consistently show that water transport through lipid membranes in the lamellar fluid phase is remarkably fast, typically occurring within seconds or less, with permeability values in the range 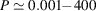
 µm s^−1^ (Dawson, 1988 ▸; Engelbert & Lawaczeck, 1985*a* ▸,*b* ▸; Hanai & Haydon, 1966 ▸; Finkelstein, 1976 ▸; Lawaczeck, 1979 ▸). These variations are primarily attributed to differences in experimental techniques and sample preparation, as well as membrane lipid composition. The predominant experimental approach in the field involves inducing rapid external osmotic stress, typically by creating salt gradients between the vesicle lumen and its exterior, and subsequently monitoring the resulting volume changes as the system equilibrates (Huster *et al.*, 1997 ▸; Finkelstein, 1976 ▸; Reeves & Dowben, 1970 ▸; Ye & Verkman, 1989 ▸; Carruthers & Melchior, 1983 ▸; Frallicciardi *et al.*, 2022*a* ▸). However, Jansen & Blume (1995 ▸) observed a significant discrepancy in water-permeability measurements between stress-free membranes and those subjected to osmotic stress, with permeability in the latter being up to an order of magnitude higher. This difference was attributed to the mechanism of water permeation: in osmotically stressed membranes, water is thought to diffuse through transient pores, whereas in stress-free membranes, permeation occurs via a simple diffusion process. More recently, a study highlighted the challenges in producing true unilamellar vesicles (Scott *et al.*, 2019 ▸), suggesting that internal vesicles or bilayer shells in extruded lipid vesicles may have influenced earlier reports of stress-free permeability data.

Various molecular-dynamics (MD) simulations have primarily focused on elucidating the mechanisms underlying passive transport (Frallicciardi *et al.*, 2022*b* ▸; Marrink & Berendsen, 1994 ▸, 1996 ▸; Venable *et al.*, 2019 ▸; Saito & Shinoda, 2011 ▸). Venable *et al.* (2019 ▸) compared experimental and computational permeability data, revealing discrepancies of up to an order of magnitude, likely attributable to the effects of the experimentally applied osmotic stress protocols. More recently, an MD study investigated the impact of membrane curvature on fluid bilayers composed of dipalmitoylphos­phatidylcholine (DPPC), demonstrating a notable increase in permeability in highly curved bilayers (Davoudi & Ghysels, 2023 ▸). Large-scale analyses utilizing simulations from the NMRlipids databank suggested non-linear dependencies of water permeation on membrane area per lipid and thickness, and overestimated permeation coefficients in simulations when compared with experiments for membranes in liquid state (Kiirikki *et al.*, 2024 ▸).

Taken together, the discrepancies between experimental and computational permeability values, along with potential experimental artefacts arising from external osmotic stress (Jansen & Blume, 1995 ▸) and/or contributions from internal shells or vesicles in extruded lipid vesicles (Scott *et al.*, 2019 ▸), highlight the need for a technique that eliminates the influence of osmotic stress while simultaneously assessing the lamellarity and stability of the studied lipid vesicles.

To address these challenges, we utilize time-resolved small-angle neutron scattering (TR-SANS) combined with stopped-flow H
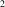
O/D
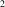
O exchange in large unilamellar vesicles (LUVs; size 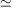 100 nm) under stress-free isotonic conditions. This approach leverages the significant scattering-length density (SLD) contrast between H
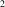
O and D
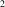
O. In this proof-of-concept study, we focus specifically on DPPC LUVs at various temperatures within the lamellar gel phase (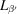). From our measurements, we derive water permeability and activation free energies for water penetration, comparing our results with existing data and analyzing MD data extracted from the NMR database (Kiirikki *et al.*, 2024 ▸). Finally, we discuss the advantages of our method as well as its potential limitations.

## 2. Materials and methods

### 2.1. Stopped-flow cell and time-resolved SANS

**Figure 1:**
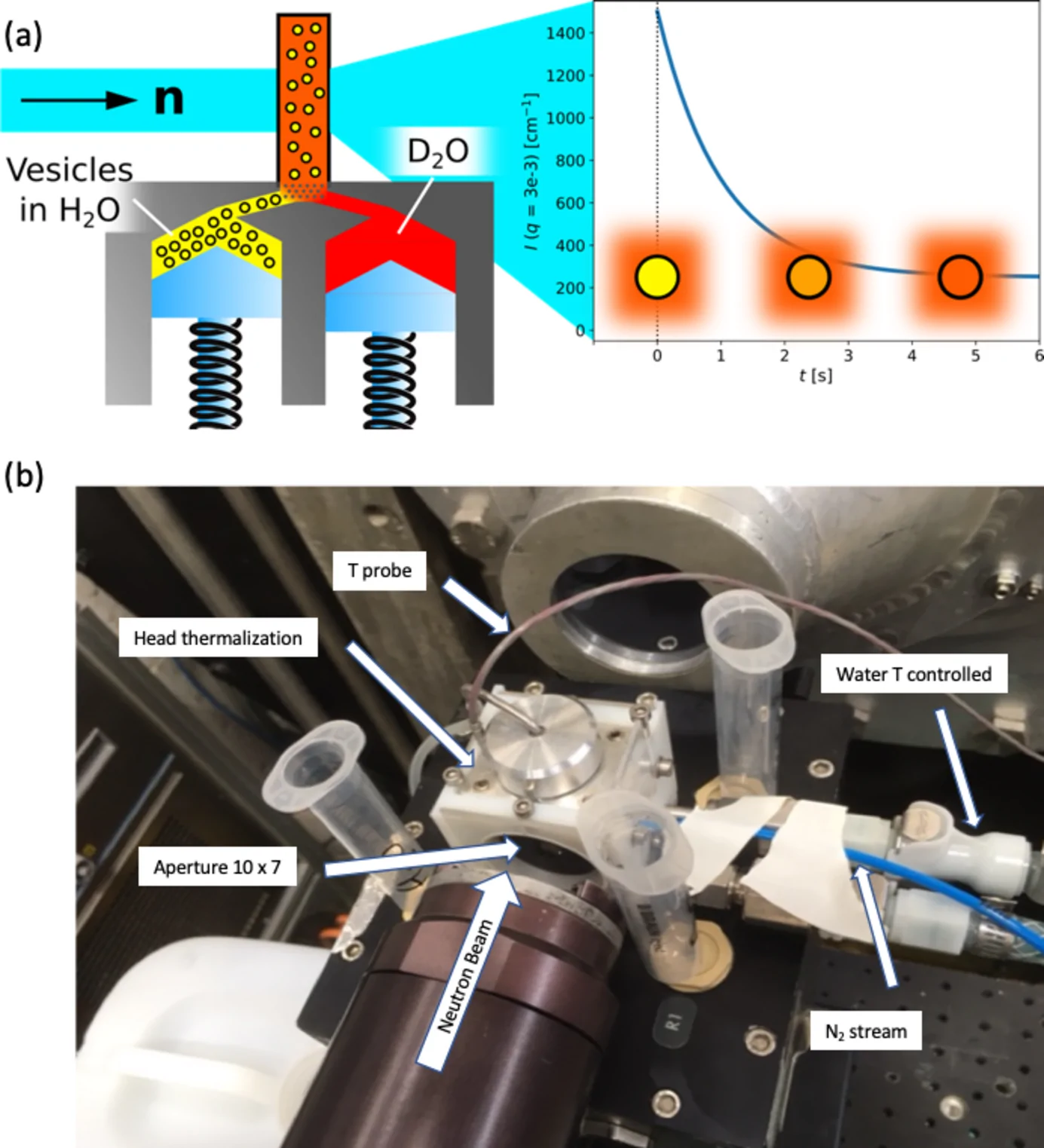
Permeability measurements by TR-SANS. (*a*) Schematic drawing of the experimental setup: D
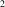
O exchange buffer and lipid vesicles in H
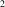
O are injected simultaneously and irradiated by a neutron beam (n). The simulated intensity decay at low *q* illustrates how the SLD in the vesicle core (yellow) gradually matches the surrounding solvent (orange) due to permeation. (*b*) Stopped-flow device mounted on the D22 SANS instrument, featuring a custom-designed small-volume head with temperature control. A nitrogen stream prevents condensation during measurements at low temperatures.

Fig. 1 ▸(*a*) illustrates the experimental setup, where lipid vesicles prepared in H
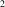
O are rapidly mixed with pure D
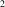
O. The permeation of D
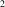
O into the vesicle lumen alters the overall contrast of the vesicles, which is monitored using TR-SANS.

To investigate fast kinetic processes, we employed the BioLogic stopped-flow module SFM-400 system, equipped with a temperature-controlled newly custom-built neutron flow-through cell designed to generate a homogeneous flow field within the rectangular cross section of the cell [Fig. 1 ▸(*b*)]. Samples were stored in thermally controlled syringes. For each measurement, 88 µL of vesicle solution (in H
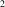
O) and 352 µL of D
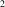
O were rapidly injected at a flow rate of 5 mL s^−1^, resulting in a final lipid concentration of 2 mg mL^−1^ in the measurement cell. Data acquisition was performed in a time-stamped ‘list mode’ and binned into 50 ms frames. The estimated dead time of 40 ms includes both the filling of the stop-flow internal lines and the time required to fill the 200 µL measurement cell at 5 mL s^−1^. Dead times of the order of a few milliseconds could be achieved by reducing the scattering volume, for example through the use of a thin-capillary geometry similar to that employed in stop-flow small-angle X-ray scattering (SAXS) measurements (Grillo, 2009 ▸). Between measurements, the measurement cell was cleaned with 6 mL of H
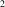
O followed by 900 µL of an H
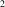
O/D
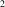
O mixture to ensure consistent initial conditions.

SANS measurements were conducted on the D22 spectrometer at the Institut Laue–Langevin (ILL), Grenoble, France. A two-detector setup was used, with the back detector centered 17.6 m from the sample and the front detector positioned off-center 1.4 m from the sample. This configuration enabled the capture of approximately two orders of magnitude in momentum transfer *q* (ranging from 0.003 to 0.6 Å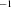) in a single measurement. The neutron beam was collimated over 17.6 m, with a wavelength of 6 Å and a spread of 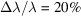 to optimize neutron flux. A sample aperture of 10 × 7 mm was directly mounted on the stop-flow head to minimize beam divergence. Data reduction was performed using *GRASP* (Dewhurst, 2023 ▸), applying flat-field, solid-angle, dead-time and transmission corrections, normalizing by incident flux, and subtracting contributions from the empty cell and solvent.

### 2.2. Preparation of liposomes

DPPC and dipalmitoylphosphatidylserine (DPPS) were purchased as powders from Avanti Polar Lipids (Alabaster, AL, USA) and used without further purification. Chloroform and methanol (pro analysis grade) were obtained from Merck KGaA, Darmstadt, Germany, and D
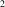
O was from Sigma–Aldrich, France. Lipid stock solutions were prepared by dissolving weighed amounts of lipids in a chloroform/methanol mixture (2:1, vol/vol) and then combining them at a molar ratio of DPPC/DPPS (95:5 mol/mol). The addition of DPPS to DPPC prevents vesicle fusion, internal vesicle formation and multilamellarity (Scott *et al.*, 2019 ▸), which is essential for maintaining stable unilamellar vesicles during rapid mixing. The organic solvent was removed from the lipid mixture by evaporation under a gentle N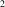 stream, followed by overnight storage in a vacuum chamber.

The resulting dry lipid films were hydrated with ultrapure H
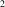
O to a final concentration of 10 mg mL^−1^ and equilibrated for one hour at a temperature 10
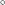
C above the transition temperature (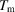 = 42
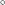
C) (Tristram-Nagle *et al.*, 1987 ▸; Nagle & Wilkinson, 1982 ▸). This was followed by five freeze–thaw cycles using dry ice, with intermittent vortex mixing. LUVs were then prepared by performing at least 31 extrusions at 50
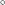
C using a handheld mini-extruder (Avanti Polar Lipids, Alabaster, AL, USA) equipped with a 100 nm pore diameter polycarbonate filter. Vesicle size and polydispersity were monitored via dynamic light scattering using a Zetasizer NANO ZS90 (Malvern Panalytical, Malvern, UK) to confirm successful extrusion.

### Modeling of H
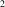
O/D
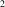
O exchange in unilamellar vesicles

2.3.

The flux *j* of water into or out of the lumen of unilamellar vesicles is directly proportional to the concentration gradient, 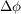, of D
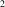
O (or H
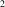
O) between the extravesicular solvent (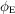) and the vesicle core (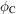): 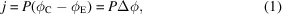
where *P* denotes the membrane permeability (Paula & Deamer, 1999 ▸).

The time evolution of 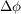 is governed by a differential equation in which the rate of change of D
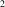
O concentration inside the vesicle is proportional to the total flux through the membrane surface *S* and inversely proportional to the vesicle volume *V*. Given that the mean vesicle radius (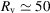
 nm) results in a lumen volume that constitutes less than 1% of the total solvent volume in the sample, we can assume that 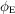 remains constant during H
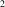
O/D
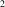
O exchange. As a result, 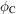 is the only time-dependent variable. For spherical vesicles, the relationship becomes 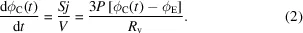
Applying the boundary conditions 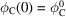 and 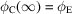, the solution for 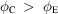 is 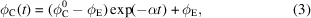
where 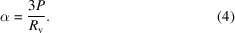
The permeability-driven change in D
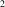
O (or H
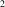
O) content within the vesicle lumen translates directly to a variation in SANS intensity. In the simplest model, the vesicle is treated as a spherical core–shell particle – with a time-dependent core SLD, 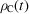, and a single membrane shell of constant SLD 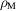, corresponding to the average SLD of the lipid bilayer (including headgroups and tails). The wavelength spread and time resolution of the experiment do not justify the use of more highly resolved SLD descriptions for the lipid bilayer (Semeraro *et al.*, 2024 ▸).

The total form factor 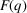, therefore, consists of contributions from both the lipid membrane (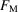) and the vesicle core (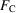), each dependent on the vesicle radius 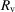 and membrane thickness 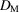: 
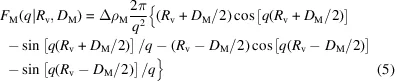
and
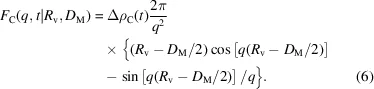
Here, 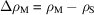 and 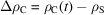 denote the contrast SLDs between the membrane/core and the external solvent (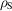, *i.e.* D
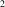
O/H
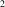
O). Upon equilibration of the external and internal solvent, 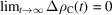, causing the core’s contribution to the overall form factor, 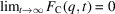, to vanish.

Accounting for polydispersity in vesicle radius with a normal distribution 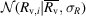, the scattering intensity is 
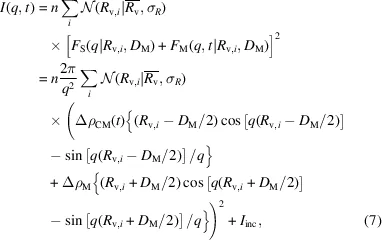
where *n* is the particle density, 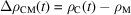 is the SLD contrast between the vesicle lumen and the lipid membrane, and 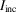 is the incoherent scattered intensity. The most significant changes are expected at low *q* [*i.e.* in the forward scattering limit, 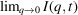]. However, for a more robust data analysis, a wider range of *q* values can be considered.

**Figure 2:**
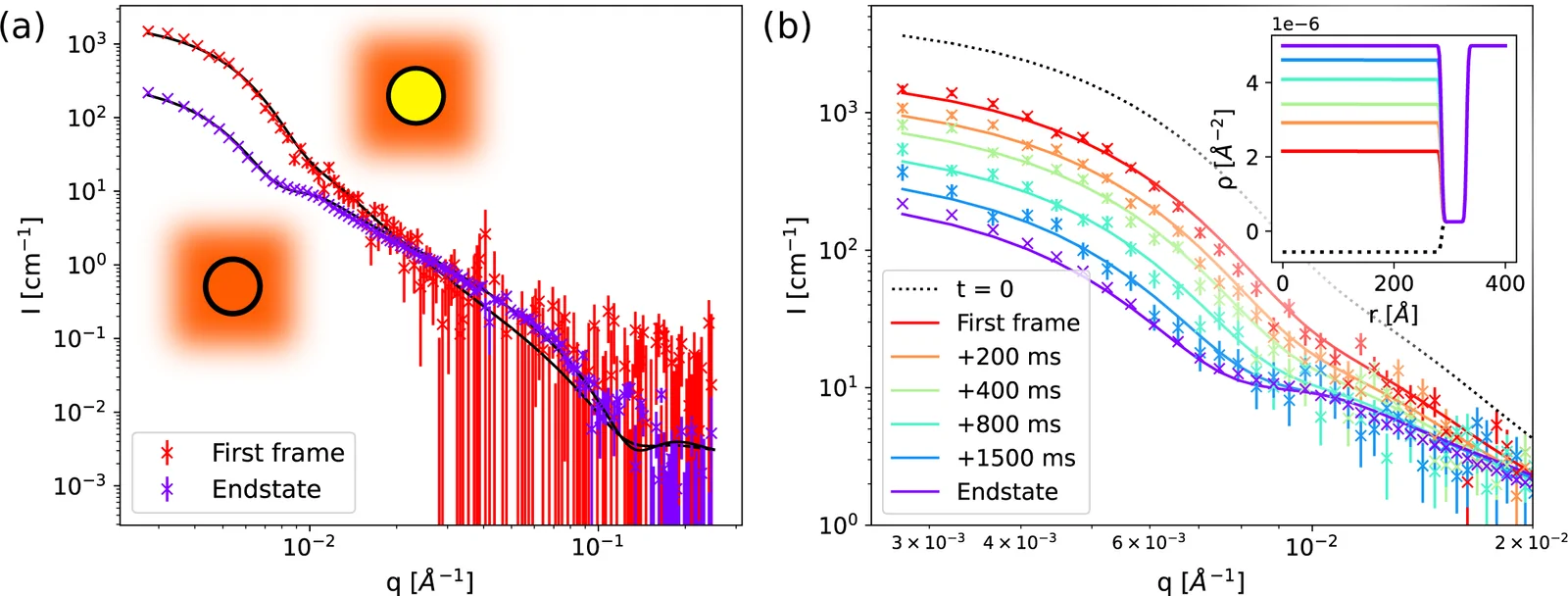
Permeability-induced changes in SANS data for DPPC LUVs at 283 K. (*a*) Comparison of initial and equilibrium scattering patterns. The first frame was recorded after a dead time of 40 ms. Solid lines represent best fits with equation (7), differing only in core SLD (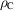). (*b*) Low-*q* scattering profiles at selected time points, with corresponding fits (solid lines). The dotted line corresponds to the calculated scattering intensity of 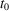. The inset displays SLD profiles for each time point.

Experimentally, we observe substantial changes in scattering intensity for 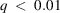
 Å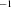 (Fig. 2 ▸). In this *q* range, 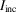 is negligible compared with the coherent signal, allowing calculation of the integral low-*q* form factor 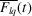 from the scattering intensity: 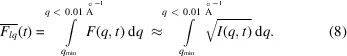
Inspection of equation (7) indicates that 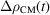 can be factored out of the integration over the scattered intensity, so that 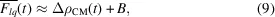
where *B* is a constant. Furthermore, 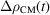 is directly related to the D
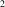
O content in the vesicle core, and therefore to the membrane permeability [*cf*. equation (3)]: 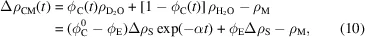
where 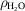 and 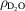 are the SLDs of H
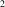
O and D
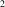
O, respectively, and 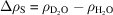.

Thus, for the analysis of our time-resolved data, we obtain 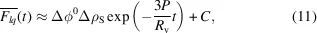
where 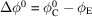 and *C* is a constant.

## 3. Results

Fig. 2 ▸(*a*) presents a comparison of the scattering intensities at 283 K for DPPC LUVs prepared in H
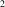
O, measured immediately after mixing with D
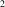
O and after full equilibration 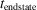. The most pronounced differences between the scattering patterns appear at low *q*, while the patterns essentially overlap at high *q*. The large error bars of the first frame at high *q* stem from the short exposure time (six repeats of 50 ms acquisition), whereas the improved statistics for the 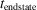 result from a longer acquisition time (5 min). The dataset of the endstate is well described by equation (7) using 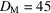
 Å, which is consistent with the previously reported membrane thickness of DPPC bilayers in the 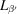 phase (Nagle & Tristram-Nagle, 2000 ▸). Parameter optimization by a least-squares fit results in a vesicle radius 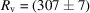
 Å with 30% polydispersity. The curve obtained for the first frame is described with the same model, varying solely the D
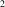
O/H
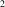
O ratio inside the vesicles. From the obtained 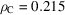
 Å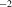, we infer that the vesicle core contains 39.1% 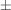 0.2% D
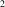
O. The excellent agreement between the model and data in the low-*q* region suggests that the overall structure of the vesicle is unperturbed by the diffusion process.

The evolution of the SANS patterns throughout the entire stopped-flow experiment is shown in Fig. 2 ▸(*b*) for 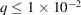
 Å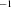, highlighting selected time frames. Analysis of the data using equation (7) reveals a progressive decrease in the contrast between the vesicle core and the surrounding H
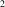
O/D
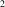
O mixture (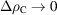), while both the membrane thickness and vesicle radius remain unchanged. This provides clear evidence that the experimental protocol preserves the vesicle structure throughout the measurement.

**Figure 3:**
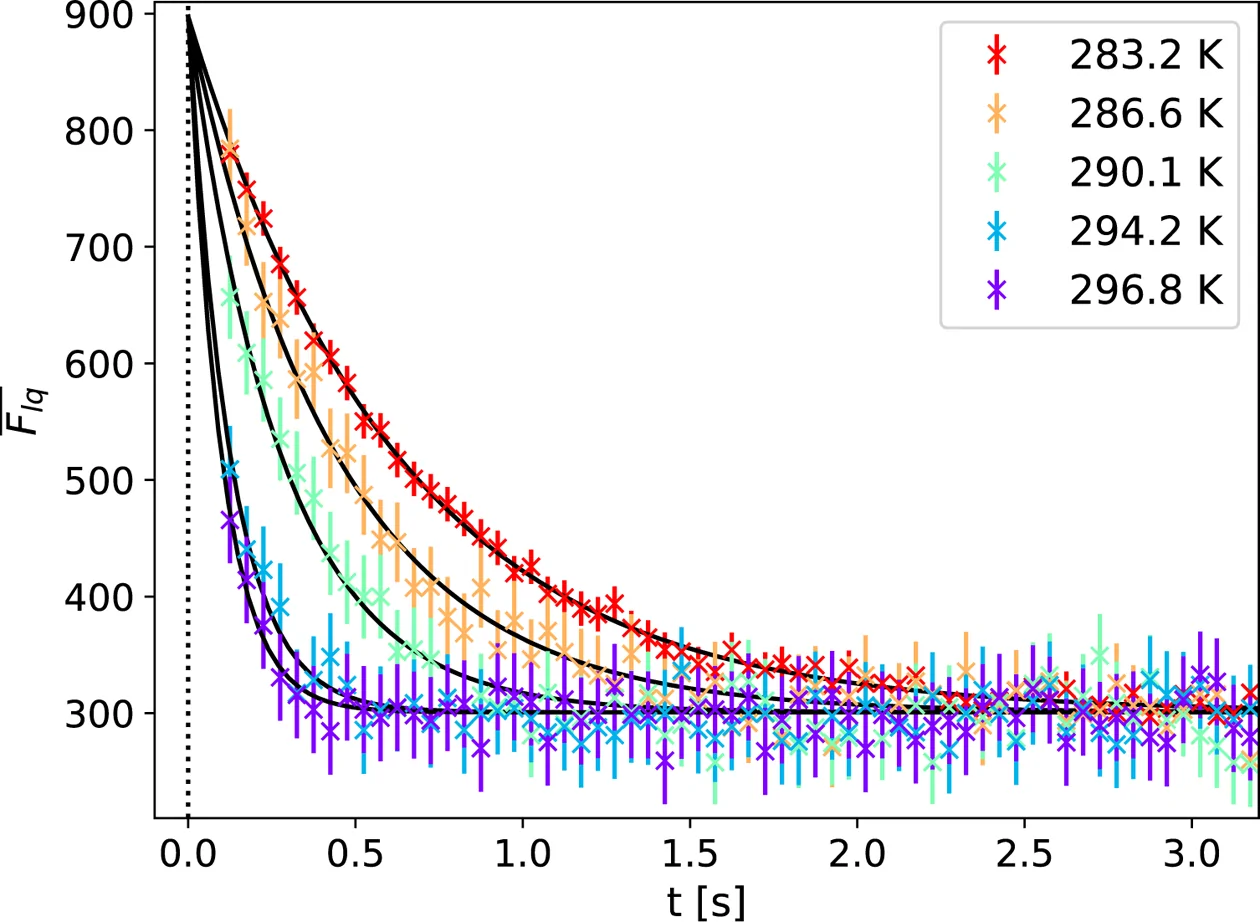
Decay of the integral low-*q* form factor at different temperatures. Black lines indicate fits using equation (11), constrained to common initial and final values for all temperatures.

The kinetics of water diffusion into the vesicles are most effectively tracked using the integral low-*q* form factor, as illustrated in Fig. 3 ▸ for various temperatures. At 283 K, 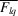 decayed to a constant value within 2.5 s. As the temperature increased, the rate of decline in 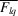 accelerated, reaching a timescale of only a few milliseconds at 297 K. For temperatures above 297 K, the kinetics were too rapid to be resolved by the experimental setup. Importantly, and consistent with the 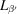 phase, the LUV structure remained unchanged across the entire temperature range accessible for the permeability measurements.

**Figure 4:**
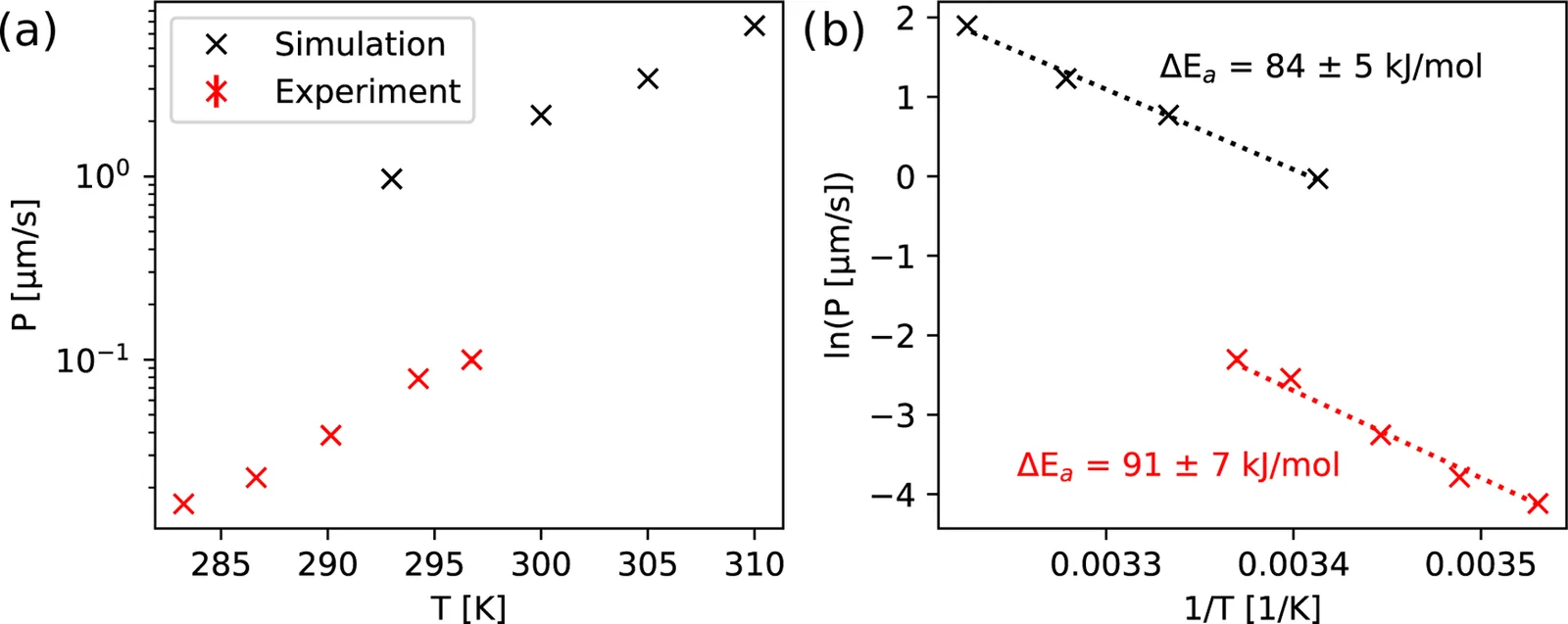
(*a*) Experimental and simulated water-permeability values for DPPC as a function of temperature. Simulation values include the average of two Slipids simulations at 293 K (NMRlipids IDs 247 and 46) (Javanainen, 2017 ▸), an Slipids simulation at 300 K (ID 542) (Javanainen *et al.*, 2017 ▸), a CHARMM36 simulation at 305 K (ID 406) (Javanainen *et al.*, 2019 ▸) and an Slipids simulation at 310 K (ID 567) (Javanainen *et al.*, 2017 ▸). (*b*) Activation energy for water permeability in DPPC, extracted from experimental (red) and simulation (black) data.

Water permeability was determined from the data in Fig. 3 ▸ using equation (11). As shown in Fig. 4 ▸(*a*), *P* increases monotonically from 0.016 µm s^−1^ at 283 K to 0.1 µm s^−1^ at 297 K. Additionally, the activation free energy barrier for water entry into the membrane was determined using the Arrhenius equation, 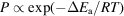, where *R* is the universal gas constant. This analysis yielded an activation energy of 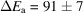
 kJ mol^−1^, as illustrated in Fig. 4 ▸(*b*).

For comparison, we extracted the temperature dependence of water-permeability MD simulations with temperatures below the main phase transition available in the NMRlipids databank (five simulations in total) (Kiirikki *et al.*, 2024 ▸), by counting the translocation events of water molecules through the bilayers (Camilo *et al.*, 2022 ▸). However, there is limited temperature overlap between the simulations and our experimental data (Fig. 4 ▸). Furthermore, the permeability values obtained from simulations are about an order of magnitude higher than those measured experimentally, although the activation energies derived from the simulations are in good agreement with the experimental values within the margin of uncertainty.

## 4. Discussion

To avoid artefacts associated with the application of external osmotic stress (Jansen & Blume, 1995 ▸), we combined stopped-flow experiments with TR-SANS to measure water permeability through bilayer lipid vesicles. Two key advantages of SANS are exploited in this approach. First, the strong SLD contrast between H
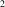
O and D
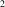
O provides high sensitivity to H
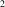
O/D
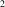
O concentration gradients across the membrane, enabling quantitative analysis of water diffusion through the bilayer. Although the present study focuses on monitoring the net influx of D
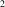
O into LUVs initially filled with H
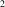
O, a reversed configuration – mixing LUVs containing D
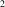
O with H
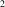
O – would be equally feasible. Second, SANS offers high sensitivity to structural parameters of the vesicles: changes in membrane thickness or lamellarity are readily detected. For instance, the appearance of Bragg peaks in the scattering profile indicates additional bilayers within the LUVs. In the present experiments, unilamellarity was ensured by incorporating 5 mol% of the negatively charged lipid DPPS into the DPPC matrix (Scott *et al.*, 2019 ▸).

The impact of vesicle lamellarity is highlighted by comparison with previous results obtained under stress-free conditions. Earlier measurements utilized either changes in refractive index detected by light scattering during H
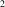
O/D
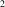
O exchange (Lawaczeck, 1979 ▸, 1984 ▸; Engelbert & Lawaczeck, 1985*a* ▸) or fluorescence quenching of a probe encapsulated within the vesicles (Lawaczeck, 1978 ▸), but lacked a direct structural characterization of the vesicles. For extruded or sonicated DPPC vesicles, permeabilities of 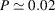
 µm s^−1^ were reported between 293 and 311 K (Jansen & Blume, 1995 ▸), while 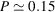
 µm s^−1^ was reported at 311 K (Engelbert & Lawaczeck, 1985*a* ▸). The elevated permeability at higher temperature may be attributable to a phase transition at 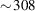
 K from 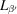 to 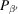 (Koynova & Caffrey, 1998 ▸; Nagle & Wilkinson, 1982 ▸; Tristram-Nagle *et al.*, 1987 ▸). In contrast, our data show a steady increase in permeability within the 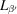 phase itself [Fig. 4 ▸(*a*)], reaching approximately 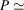
0.07 µm s^−1^ at 293 K. This value indicates that in our setup water permeates DPPC bilayers up to 50 times faster than previously reported. Although a direct comparison using identical conditions was not performed, it is likely that the lower permeabilities in earlier studies result from the presence of multilamellar vesicles or small entrapped vesicles, which substantially hinder water diffusion.

Nevertheless, we cannot entirely rule out the presence of artefacts in our measurements. Notably, LUVs are known to exhibit faceted rather than perfectly spherical surfaces in the lamellar gel phase (Jiménez-Rojo *et al.*, 2015 ▸), which may indicate the presence of structural defects. Such defects will enhance permeation (Xiang & Anderson, 1994 ▸) and thus increase the apparent permeability. Ideally, measurements should be performed above the main phase transition, in the fluid 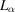 phase, where such defects are minimized. However, in the fluid phase, water translocation occurs too rapidly to capture with the current experimental setup.

MD simulations provide valuable insights into water permeability through defect-free bilayers. For fluid DPPC membranes, permeabilities of 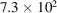
 µm s^−1^ at 350 K (Marrink & Berendsen, 1994 ▸) and 50 µm s^−1^ at 323 K (Davoudi & Ghysels, 2023 ▸) have been reported.

In this work, *P* values for gel-phase DPPC were obtained by analyzing permeation events in MD data from the NMRlipids databank (Kiirikki *et al.*, 2024 ▸) using established methodologies (Camilo *et al.*, 2022 ▸). Interestingly, these simulated gel-phase permeabilities exceed our experimental results by approximately an order of magnitude [Fig. 4 ▸(*a*)]. However, the activation energies for water permeation derived from both experiment and simulation agree within the respective uncertainties [Fig. 4 ▸(*b*)].

These results indicate that, while MD simulations accurately capture the fundamental mechanisms of water permeation, the resulting absolute permeability values remain sensitive to subtle force-field limitations and simulation parameters. Overestimation of water permeation was also observed in large-scale analyses from the NMRlipids databank for membranes in liquid state (Kiirikki *et al.*, 2024 ▸). One plausible explanation for these observations is the overestimated self-diffusion coefficient for the TIP3P water model (often used in lipid-bilayer MD simulations, including simulations analyzed here), which leads, for example, to overestimation of protein rotational dynamics in MD simulations (Ollila *et al.*, 2018 ▸). However, we cannot fully exclude the effects that could arise from possibly inaccurate description of gel-phase structure in MD simulations. Integrating the present experimental methodology with advanced structural characterization, such as joint SAXS/SANS measurements (Semeraro *et al.*, 2024 ▸), promises a more rigorous foundation for identifying the membrane characteristics that regulate permeation. Such an approach would also facilitate refinement of MD force fields and simulation protocols, ultimately advancing the quantitative and mechanistic understanding of the factors that govern diffusive transport of water and other solutes across lipid membranes.

## 5. Conclusions

This study establishes proof of concept that TR-SANS, combined with rapid contrast variation, provides a robust non-invasive means to quantify water permeation in gel-phase lipid vesicles under stress-free conditions, thereby closely reflecting native membrane transport processes. A distinctive strength of this approach lies in its unique ability to concurrently monitor both transmembrane water flux and membrane structure. This is particularly significant given the pronounced dependence of diffusive water permeability on membrane composition, lipid packing and thermotropic phase state.

The results presented here naturally motivate extending this methodology to encompass a wider range of lipid compositions, solutes and temperature conditions. Such systematic studies will provide deeper insight into the fundamental determinants of diffusive solute transport across biological membrane mimics. Advancing this understanding will not only enrich the field of membrane biophysics but also inform the design of drug delivery systems and the engineering of high-performance biomimetic materials.
